# Supplementary material for: Circulating miRNA-451a and miRNA-328-3p as Potential Markers of Coronary Artery Aneurysmal Disease
Source: Int J Mol Sci. 2023 Mar 18;24(6):5817. doi: 10.3390/ijms24065817 (PMC10058788; doi:10.3390/ijms24065817)
Supplement: Supplementary file 1 [file ijms-24-05817-s001.zip › ijms-2287080-supplementary.pdf]

Table S1. Genes regulated by hsa-miR-23a-3p

Gene symbol	Interpretation
**TSC1**	Homo sapiens TSC complex subunit 1 (TSC1), transcript variant 1,mRNA.
**BRWD1**	Homo sapiens bromodomain and WD repeat domain containing 1 (BRWD1),transcript variant 3, mRNA.
**TMEM64**	Homo sapiens transmembrane protein 64 (TMEM64), transcript variant1, mRNA.
**DNAJC21**	Homo sapiens DnaJ heat shock protein family (Hsp40) member C21(DNAJC21), transcript variant 2, mRNA.
**ATAT1**	Homo sapiens alpha tubulin acetyltransferase 1 (ATAT1), transcript variant 1, mRNA.
**TPM3**	Homo sapiens tropomyosin 3 (TPM3), transcript variant Tpm3.2, mRNA.
**ZNF275**	Homo sapiens zinc finger protein 275 (ZNF275), transcript variant2, mRNA.
**PLAG1**	Homo sapiens PLAG1 zinc finger (PLAG1), transcript variant 2, mRNA.
**FSD1L**	Homo sapiens fibronectin type III and SPRY domain containing 1 like(FSD1L), transcript variant 3, mRNA.
**SLC6A15**	Homo sapiens solute carrier family 6 member 15 (SLC6A15),transcript variant 3, mRNA.
**ZNF268**	Homo sapiens zinc finger protein 268 (ZNF268), transcript variant2, mRNA.
**ZNF701**	Homo sapiens zinc finger protein 701 (ZNF701), transcript variant1, mRNA.
**GGA3**	Homo sapiens Golgi associated, gamma adaptin ear containing, ARF binding protein 3 (GGA3), transcript variant 1, mRNA.
**CD302**	Homo sapiens CD302 molecule (CD302), transcript variant 2, mRNA.
**SEMA6D**	Homo sapiens semaphoring 6D (SEMA6D), transcript variant 7, mRNA.
**PIK3R1**	Homo sapiens phosphoinositide-3-kinase regulatory subunit 1(PIK3R1), transcript variant 4, mRNA.
**MYH10**	Homo sapiens myosin heavy chain 10 (MYH10), transcript variant 1,mRNA.
**GLS**	Homo sapiens glutaminase (GLS), transcript variant 2, mRNA; nuclear gene for mitochondrial product.
**SDHD**	Homo sapiens succinate dehydrogenase complex subunit D (SDHD),transcript variant 2, mRNA; nuclear gene for mitochondrial product.
**GRTP1**	Homo sapiens growth hormone regulated TBC protein 1 (GRTP1),transcript variant 2, mRNA.
**MEM170A**	Homo sapiens transmembrane protein 170A (TMEM170A), transcript variant 2, mRNA.
**ST7L**	Homo sapiens suppression of tumorigenicity 7 like (ST7L),transcript variant 7, mRNA.
**MMGT1**	Homo sapiens membrane magnesium transporter 1 (MMGT1), transcript variant 2, mRNA.
**PPARGC1A**	Homo sapiens PPARG coactivator 1 alpha (PPARGC1A), transcript variant 1, mRNA.
**MGAT5**	Homo sapiens alpha-1,6-mannosylglycoprotein6-beta-N-acetylglucosaminyltransferase (MGAT5), transcript variant
**FOXO3**	Homo sapiens forkhead box O3 (FOXO3), transcript variant 1, mRNA.
**FUT4**	Homo sapiens fucosyltransferase 4 (FUT4), mRNA.
**SOCS6**	Homo sapiens suppressor of cytokine signaling 6 (SOCS6), mRNA.
**TERF2**	Homo sapiens telomeric repeat binding factor 2 (TERF2), mRNA.
**UQCRFS1**	Homo sapiens ubiquinol-cytochrome c reductase, Rieske iron-sulfurpolypeptide 1 (UQCRFS1), mRNA; nuclear gene for mitochondrial
**LMAN2**	Homo sapiens lectin, mannose binding 2 (LMAN2), mRNA.
**RNF38**	Homo sapiens ring finger protein 38 (RNF38), transcript variant 1,mRNA.

**DYNLL2**	Homo sapiens dynein light chain LC8-type 2 (DYNLL2), mRNA.
**STT3B**	Homo sapiens STT3 oligosaccharyltransferase complex catalytic subunit B (STT3B), mRNA.
**SPTY2D1**	Homo sapiens SPT2 chromatin protein domain containing 1 (SPTY2D1),mRNA.

Table S2. Genes regulated by hsa-miR-210-3p

Gene symbol	Interpretation
**RPL22**	Homo sapiens ribosomal protein L22 (RPL22), mRNA.
**MITF**	Homo sapiens melanocyte inducing transcription factor (MITF),transcript variant 4, mRNA.
**FGFRL1**	Homo sapiens fibroblast growth factor receptor like 1 (FGFRL1),transcript variant 1, mRNA.
**GTDC1**	Homo sapiens glycosyltransferase like domain containing 1 (GTDC1),transcript variant 1, mRNA.
**U2AF2**	Homo sapiens U2 small nuclear RNA auxiliary factor 2 (U2AF2),transcript variant 2, mRNA.
**SEH1L**	Homo sapiens SEH1 like nucleoporin (SEH1L), transcript variant 1,mRNA.
**SCN1B**	Homo sapiens sodium voltage-gated channel beta subunit 1 (SCN1B),transcript variant a, mRNA.
**HMGCS1**	Homo sapiens 3-hydroxy-3-methylglutaryl-CoA synthase 1 (HMGCS1),transcript variant 1, mRNA.
**FOXP3**	Homo sapiens forkhead box P3 (FOXP3), transcript variant 2, mRNA.
**CBX1**	Homo sapiens chromobox 1 (CBX1), transcript variant 2, mRNA.
**TFRC**	Homo sapiens transferrin receptor (TFRC), transcript variant 2,mRNA.
**DDAH1**	Homo sapiens dimethylarginine dimethylaminohydrolase 1 (DDAH1),transcript variant 2, mRNA.
**GIT2**	Homo sapiens GIT ArfGAP 2 (GIT2), transcript variant 6, mRNA.
**ATG7**	Homo sapiens autophagy related 7 (ATG7), transcript variant 2,mRNA.
**RCC2**	Homo sapiens regulator of chromosome condensation 2 (RCC2),transcript variant 2, mRNA.
**VAMP4**	Homo sapiens vesicle associated membrane protein 4 (VAMP4),transcript variant 2, mRNA.
**RUNX1T1**	Homo sapiens RUNX1 partner transcriptional co-repressor 1(RUNX1T1), transcript variant 5, mRNA.
**NFIC**	Homo sapiens nuclear factor I C (NFIC), transcript variant 1, mRNA.
**TP53I11**	Homo sapiens tumor protein p53 inducible protein 11 (TP53I11),transcript variant 1, mRNA.
**ERP27**	Homo sapiens endoplasmic reticulum protein 27 (ERP27), transcript variant 2, mRNA.
**ZNF618**	Homo sapiens zinc finger protein 618 (ZNF618), transcript variant2, mRNA.
**VMP1**	Homo sapiens vacuole membrane protein 1 (VMP1), transcript variant2, mRNA.
**MRE11**	Homo sapiens MRE11 homolog, double strand break repair nuclease(MRE11), transcript variant 3, mRNA.
**DIMT1**	Homo sapiens DIMT1 rRNA methyltransferase and ribosome maturation factor (DIMT1), transcript variant 2, mRNA.
**CNTNAP5**	Homo sapiens contactin associated protein family member 5(CNTNAP5), transcript variant 3, mRNA.
**KCNJ6**	Homo sapiens potassium inwardly rectifying channel subfamily Jmember 6 (KCNJ6), mRNA.

**NPTX1**	Homo sapiens neuronal pentraxin 1 (NPTX1), mRNA.
**DYRK2**	Homo sapiens dual specificity tyrosine phosphorylation regulated kinase 2 (DYRK2), transcript variant 1, mRNA.
**HOXA1**	Homo sapiens homeobox A1 (HOXA1), transcript variant 1, mRNA.
**ACTR1A**	Homo sapiens actin related protein 1A (ACTR1A), mRNA.
**PKIA**	Homo sapiens cAMP-dependent protein kinase inhibitor alpha (PKIA),transcript variant 1, mRNA.
**UBQLN1**	Homo sapiens ubiquilin 1 (UBQLN1), transcript variant 1, mRNA.
**EHD2**	Homo sapiens EH domain containing 2 (EHD2), mRNA.
**SERTAD2**	Homo sapiens SERTA domain containing 2 (SERTAD2), mRNA.
**HECTD1**	Homo sapiens HECT domain E3 ubiquitin protein ligase 1 (HECTD1),mRNA.
**MNT**	Homo sapiens MAX network transcriptional repressor (MNT), mRNA.
**HIF3A**	Homo sapiens hypoxia inducible factor 3 subunit alpha (HIF3A),transcript variant 2, mRNA.

Table S3. Genes regulated by hsa-miR-328-3p

Gene symbol	Interpretation
**HNRNPL**	Homo sapiens heterogeneous nuclear ribonucleoprotein L (HNRNPL),transcript variant 2, mRNA.
**NDUFV3**	Homo sapiens NADH: ubiquinone oxidoreductase subunit V3 (NDUFV3),transcript variant 2, mRNA; nuclear gene for mitochondrial product.
**SKA1**	Homo sapiens spindle and kinetochore associated complex subunit 1(SKA1), transcript variant 1, mRNA.
**UBTF**	Homo sapiens upstream binding transcription factor (UBTF),transcript variant 2, mRNA.
**PTPRJ**	Homo sapiens protein tyrosine phosphatase receptor type J (PTPRJ),transcript variant 2, mRNA.
**EZR**	Homo sapiens ezrin (EZR), transcript variant 2, mRNA.
**ARHGEF25**	Homo sapiens Rho guanine nucleotide exchange factor 25 (ARHGEF25),transcript variant 3, mRNA.
**SZRD1**	Homo sapiens SUZ RNA binding domain containing 1 (SZRD1),transcript variant 1, mRNA.
**ORMDL1**	Homo sapiens ORMDL sphingolipid biosynthesis regulator 1 (ORMDL1),transcript variant 2, mRNA.
**MAP4**	Homo sapiens microtubule associated protein 4 (MAP4), transcript variant 4, mRNA.
**OTUD5**	Homo sapiens OTU deubiquitinase 5 (OTUD5), transcript variant 2,mRNA.
**ETV3**	Homo sapiens ETS variant transcription factor 3 (ETV3), transcript variant 1, mRNA.
**FKBP5**	Homo sapiens FKBP prolyl isomerase 5 (FKBP5), transcript variant 2,mRNA.
**MKI67**	Homo sapiens marker of proliferation Ki-67 (MKI67), transcript variant 2, mRNA.
**TYW3**	Homo sapiens tRNA-yW synthesizing protein 3 homolog (TYW3),transcript variant 2, mRNA.
**HSDL2**	Homo sapiens hydroxysteroid dehydrogenase like 2 (HSDL2),transcript variant 2, mRNA.
**BACE1**	Homo sapiens beta-secretase 1 (BACE1), transcript variant e, mRNA.
**SNX12**	Homo sapiens sorting nexin 12 (SNX12), transcript variant 1, mRNA.
**POM121**	Homo sapiens POM121 transmembrane nucleoporin (POM121), transcript variant 1, mRNA.

**STT3A**	Homo sapiens STT3 oligosaccharyltransferase complex catalyticsubunit A (STT3A), transcript variant 1, mRNA.
**SMC1A**	Homo sapiens structural maintenance of chromosomes 1A (SMC1A),transcript variant 2, mRNA.
**RALGAPB**	Homo sapiens Ral GTPase activating protein non-catalytic subunitbeta (RALGAPB), transcript variant 2, mRNA.
**TMEM132B**	Homo sapiens transmembrane protein 132B (TMEM132B), transcript variant 2, mRNA.
**CBX6**	Homo sapiens chromobox 6 (CBX6), transcript variant 2, mRNA.
**HMGB1**	Homo sapiens high mobility group box 1 (HMGB1), transcript variant3, mRNA.
**AGO1**	Homo sapiens argonaute RISC component 1 (AGO1), transcript variant1, mRNA.
**KCNK6**	Homo sapiens potassium two pore domain channel subfamily K member 6(KCNK6), mRNA.
**SCD**	Homo sapiens stearoyl-CoA desaturase (SCD), mRNA.
**LPCAT3**	Homo sapiens lysophosphatidylcholine acyltransferase 3 (LPCAT3),mRNA.
**TMOD3**	Homo sapiens tropomodulin 3 (TMOD3), mRNA.
**TMEM33**	Homo sapiens transmembrane protein 33 (TMEM33), mRNA.

Table S4. Genes regulated by hsa-miR-425-3p

Gene symbol	Interpretation
**VAV3**	Homo sapiens vav guanine nucleotide exchange factor 3 (VAV3),transcript variant 2, mRNA.
**MECP2**	Homo sapiens methyl-CpG binding protein 2 (MECP2), transcript variant 2, mRNA.
**ISLR2**	Homo sapiens immunoglobulin superfamily containing leucine rich repeat 2 (ISLR2), transcript variant 1, mRNA.
**GABPB2**	Homo sapiens GA binding protein transcription factor subunit beta 2(GABPB2), transcript variant 3, mRNA.
**RAC1**	Homo sapiens Rac family small GTPase 1 (RAC1), transcript variantRac1, mRNA.
**SH3RF2**	Homo sapiens SH3 domain containing ring finger 2 (SH3RF2), mRNA.

Table S5. Genes regulated by hsa-miR-451a

Gene symbol		Interpretation
**hsa-miR-451a**	IL6R	Homo sapiens interleukin 6 receptor (IL6R), transcript variant 1,mRNA
